# Treat-to-target strategies for the management of familial Mediterranean Fever in children

**DOI:** 10.1186/s12969-023-00875-y

**Published:** 2023-09-26

**Authors:** Lisa Ehlers, Elisabeth Rolfes, Mareike Lieber, Dominik Müller, Elke Lainka, Faekah Gohar, Günter Klaus, Hermann Girschick, Jana Hörstermann, Jasmin Kümmerle-Deschner, Jürgen Brunner, Katharina Palm-Beden, Klaus Tenbrock, Lusine von Wrangel, Maria Faßhauer, Norbert Blank, Ralf Trauzeddel, Anne Sae Lim von Stuckrad, Sonja Higgins, Tatjana Welzel, Thomas Lutz, Véronique Hentgen, Dirk Foell, Helmut Wittkowski, Tilmann Kallinich

**Affiliations:** 1 Department of Paediatric Pulmonology, Immunology and Critical Care Medicine, Charité – Universitätsmedizin Berlin, corporate member of Freie Universität Berlin and Humboldt-Universität zu Berlin, Berlin, Germany; 2 Department of Paediatrics, Division of Gastroenterology, Nephrology and Metabolic Diseases, Charité - Universitätsmedizin Berlin, corporate member of Freie Universität Berlin and Humboldt-Universität zu Berlin, Berlin, Germany; 3 Department of Paediatrics II, University Hospital Essen, Children’s Hospital, Essen, Germany; 4 Clinic of Paediatric and Adolescent Rheumatology, St. Josef-Stift Sendenhorst, Northwest German Center for Rheumatology, Sendenhorst, Germany; 5 KfH Center of Paediatric Nephrology, Department of Paediatric Nephrology, Marburg, Germany; 6 Vivantes Klinikum Friedrichshain, Children’s Hospital, Berlin, Germany; 7 Deutsches Rheuma-Forschungszentrum (DRFZ), An Institute of the Leibniz Association, Berlin, Germany; 8 Autoinflammation Reference Center Tübingen (arcT), Division of Paediatric Rheumatology, Department of Paediatrics, University Hospital Tübingen, Tübingen, Germany; 9 Department of Paediatrics, Medical University Innsbruck, Danube Private University, Innsbruck, Krems, Austria; 10 Department of Paediatric Pneumology, Allergology and Immunology, RWTH Aachen, Aachen, Germany; 11 Patient representative, Berlin, Germany; 12 ImmunoDeficiencyCenter Leipzig (IDCL), Hospital St. Georg GmbH Leipzig, Academic Teaching Hospital of the University of Leipzig, Leipzig, Germany; 13 Department of Hematology, Oncology and Rheumatology, Internal Medicine V, University Hospital of Heidelberg, Heidelberg, Germany; 14 Department of Paediatrics, Helios Klinikum Berlin-Buch, Berlin, Germany; 15 Paediatric medical practice Hürthpark, Hürth, Germany; 16 Paediatric Pharmacology and Pharmacometrics, University Children’s Hospital Basel (UKBB), University Basel, Basel, Switzerland; 17 Center for Rheumatology, Paediatric Rheumatology, Heidelberg, Germany; 18 Department of Paediatrics, National Reference Center for Auto-inflammatory Diseases and Amyloidosis, CEREMAIA, Versailles Hospital, Versailles, France; 19 Department of Paediatric Rheumatology and Immunology, University Hospital Münster, Münster, Germany; 20 Berlin Institute of Health (BIH), Berlin, Germany

**Keywords:** Children, Colchicine, Colchicine resistance, Disease activity, Familial Mediterranean Fever, interleukin-1 antagonists, Medication adherence, Treat-to-target

## Abstract

**Background:**

The objective of this initiative was to develop a treat-to-target (T2T) approach for the management of patients with Familial Mediterranean Fever (FMF), including the definition of a complex treatment target, and establish strategies that improve patient care and long-term outcome.

**Methods:**

An initial set of statements as well as a flow chart visualising the proposed concept was developed. To adapt the preliminary statements to the current state of knowledge, a systematic literature search was performed and the modified statements were subject to a Delphi approach. To ensure the applicability of the statements in daily practice, an online survey was conducted among paediatric rheumatologists in Germany. In addition, data from the national AID-NET registry were analysed with respect to therapeutic response.

**Results:**

This T2T initiative yielded a total of 26 statements guiding FMF management with respect to diagnosis, treatment targets, treatment strategies and monitoring. The online survey identified cut-off values for inflammatory markers indicating treatment intensification and appropriate measures in case of colchicine intolerance or non-adherence. The analysis of data derived from the national AID-NET showed that colchicine therapy was successfully terminated in 61% of patients (27 out of 44) with heterozygous *MEFV* mutations. Multidimensional treatment targets incorporating objective and subjective reported outcome measures were developed. These provide the basis for stratifying patients into the following treatment paths: continue colchicine, persisting attacks / inflammation, colchicine intolerance, persisting arthritis, colchicine reduction and adjustment/reduction of biologics.

**Conclusions:**

The proposed consensus treatment plan for the management of FMF incorporates multidimensional targets allowing transparent treatment decisions, which will promote personalised disease management and increase adherence to therapy.

**Supplementary Information:**

The online version contains supplementary material available at 10.1186/s12969-023-00875-y.

## Introduction

Familial Mediterranean Fever (FMF) is the most common monogenic autoinflammatory disease [1]. It is characterised by recurrent short-lasting fever episodes accompanied by serositis and elevated inflammatory parameters [2]. Amyloidosis occurs as a long-term complication in approximately 11% of patients and represents the main cause of mortality [2, 3].

The disease is caused by mutations in the MEditerranean FeVer (*MEFV*) gene encoding pyrin, a protein involved in caspase-1 activation and interleukin (IL)-1β production [4–6]. In Germany, the incidence of FMF is 48 in 10^6^ children [7]. In children of Eastern Mediterranean origin, the prevalence is up to 18 times higher due to the carrier frequency in this area [8, 9]. Disease onset occurs during childhood in the majority of FMF patients. Affected children differ from patients with adult-onset FMF with respect to the clinical phenotype [10, 11]. Moreover, symptom severity varies with age of onset in childhood [12, 13]. This warrants the development of children-oriented treatment guidelines.

Colchicine represents the mainstay of FMF therapy and its efficacy in preventing attacks and the occurrence of amyloidosis has been shown in clinical trials and large cohort studies [14]. The continuous life-long colchicine application resolves symptoms in up to 2/3 of patients, [15] in up to 1/3 of patients this therapy significantly improves clinical symptoms and systemic inflammation [16]. In 5–10% of patients colchicine monotherapy does not lead to satisfactory disease control [16]. These patients continue to suffer from frequent severe attacks, persistent inflammation with potential long-term consequences of e.g. amyloidosis, growth retardation, decreased quality of life or depression and/or persisting arthritis [17].

The emergence of the newly approved IL-1 antagonists canakinumab and anakinra as well as experience with other drugs in specific treatment scenarios are expanding the therapeutic options in FMF [18–22]. Together with both the short-term and long-term consequences of ongoing disease activity and the challenge of defining disease activity in FMF, this highlights the need for the development of standardised and comprehensive treatment recommendations to improve patient-oriented therapy management.

The treat-to-target (T2T) concept introduces treatment escalation and de-escalation depending on the achievement of numerical therapeutic targets [23, 24]. Recently, a German multi-centre study confirmed superiority of the T2T concept to achieve remission in polyarticular juvenile idiopathic arthritis [25]. While there are existing EULAR recommendations for the management of FMF, [14] this article focuses on the establishment of a target-oriented treatment strategy and aims to complement the existing guidelines. It comprises an easy-to-use work flow to be implemented in routine clinical care.

The aim of this initiative was to develop a T2T approach for the treatment of FMF that (i) unifies the plethora of available disease activity scores in the form of a multidimensional treatment target and (ii) takes into account the recent emergence of novel therapeutic agents. The development of these strategies serves the overall purpose of improving patient care and long-term outcome.

## Methods

The compilation and adaptation of T2T strategies was performed in a multi-step approach consulting the expertise of a large group of specialists via online surveys and project group meetings as well as in-depth verification and modification in small working groups (Supplementary Figure S1).

### Steering group and statement development

The *Protokolle in der Kinderrheumatologie* (PRO-KIND) initiative of the German Society for Paediatric Rheumatology (GKJR) aims to develop consensus-based treat-to-target (T2T) strategies for a variety of rheumatic, autoimmune and autoinflammatory diseases in children. In the FMF sub-committee, a steering group of four paediatric rheumatologists (TK, VH, DF, HW) and one resident (LE) developed an initial set of statements as well as a flow chart visualising the proposed concept. The statements were adopted from existing recommendations based on previous systematic literature reviews where applicable.

### Online survey

In order to ensure the applicability of the statements in daily practice, an online survey was performed among paediatric rheumatologists in Germany prior to the generation of the original statements. The survey consisting of 21 clinical scenarios and a set of management-related questions was sent out to 150 accredited paediatric rheumatologists in Germany. The survey addressed the following topics: diagnosis (6 scenarios), treatment initiation (2 scenarios), adjustment of the colchicine dose (3 scenarios), use of biologics (8 scenarios), and treatment reduction (2 scenarios). In the survey, the level of inflammation was defined as follows: low (SAA 10–50 mg/L and/or CRP 5–20 mg/L), medium (SAA 50–100 mg/L and/or CRP 20–40 mg/L), and high (SAA > 100 mg/L and/or CRP > 40 mg/L). The following *MEFV* genotypes were covered in the clinical scenarios: clear pathogenic variants (homozygous or compound heterozygous variants [M680I, M694I, M694V, V726A]), heterozygous pathogenic variants or homozygous variants of unknown significance (E148Q).

The results of this survey were considered during the initial compilation of the draft statements and served as orientation in the subsequent consensus process.

### Literature review and statement adaptation

Twenty-seven statements were generated and modified by the steering group as described above and presented to the project group consisting of 18 paediatric rheumatologists, two paediatric nephrologists, one resident and one patient representative in May 2021. In order to adapt the preliminary statements to the current state of knowledge, search strategies for a literature review were agreed upon for each statement. The responsibility for the individual statements was assigned to members of the panel of specialists. In case of pre-existing statements, the search comprised the time period not yet covered by previous systematic literature reviews. For topics that were not included in such prior works, the search was performed without restrictions by publication date.

**Fig. 1:**
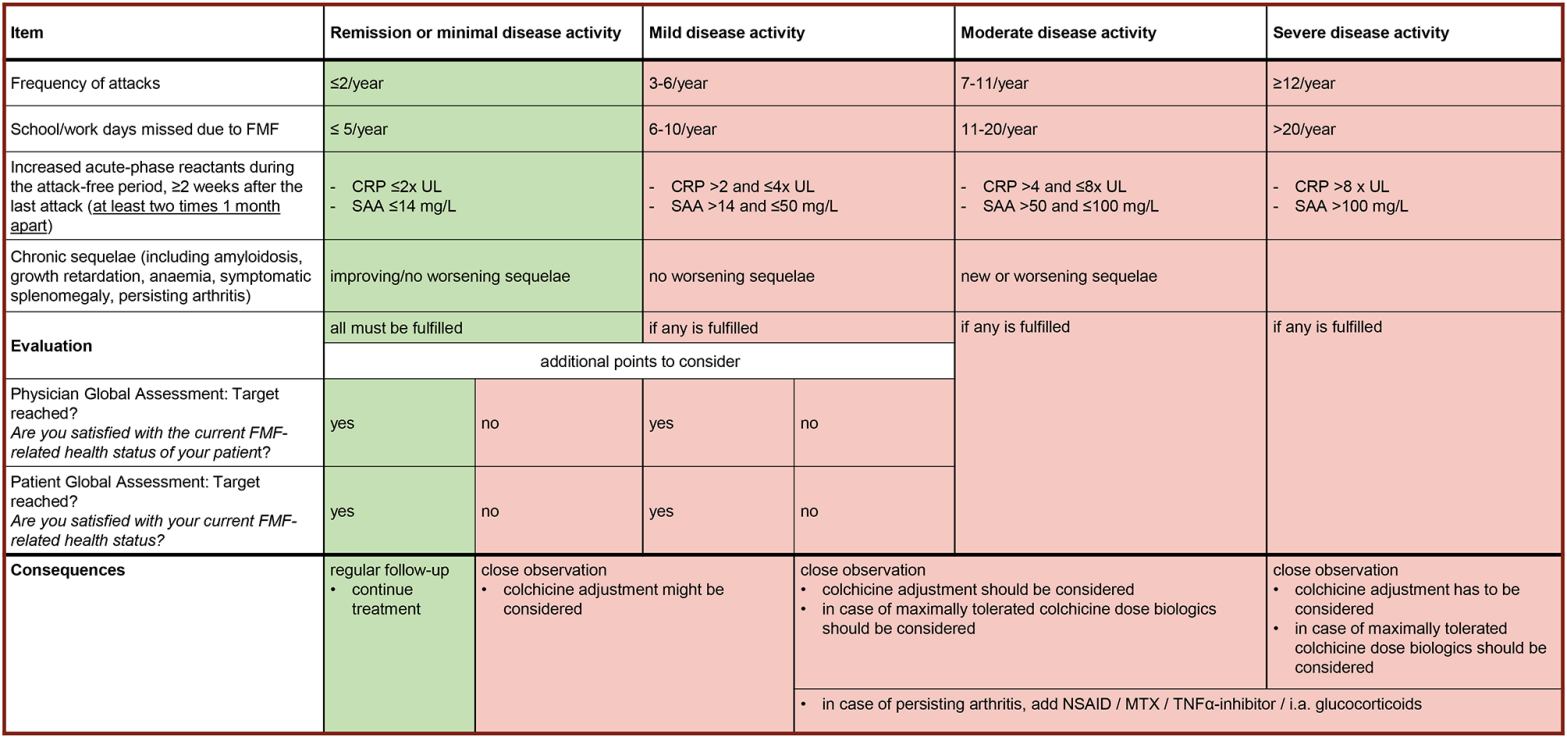
Composite score of multidimensional treatment targets to assess disease activity in patients with familial Mediterranean fever. Legend: CRP, C-reactive protein; MTX, methotrexate; NSAID, non-steroidal anti-inflammatory drug; UL, upper limit; SAA, serum amyloid A

The MEDLINE literature database was searched through PubMed in June 2021 according to the search strategies provided in Supplementary Table S1. The statements were adapted accordingly by the responsible specialists. Following these adjustments, an online poll was conducted to collect proposals for modification to be discussed during the subsequent meeting. In addition, data from the national network for autoinflammatory syndromes in children and adolescents (AID-NET) registry describing the use of colchicine in FMF were taken into account when adjusting the statements [26]. A steering group of four paediatric rheumatologists from Germany and France (DF, VH, HW, TK) established a set of treatment targets by unifying existing recommendations and disease activity scores [14, 26–31]. This process yielded a set of target criteria (Fig. 1) that was not subject to the consensus process following the Delphi method. It was however discussed and approved by the project group and found suitable as a foundation of the treat-to-target approach.

### Consensus conference

The consensus conference took place virtually on 1st and 2nd July 2021. The participants included the project group and one patient representative. The meeting was moderated by an independent host who was not eligible for voting. Consensus was achieved with the help of a Delphi approach: All statements were presented to the participants by the specialists who had conducted the respective literature review. Each participant was then given the opportunity to comment. This was followed by an anonymous voting. If the agreement was < 80%, the statement was adjusted according to the comments until a consensus of ≥ 80% was reached.

### Analysis of the national AID-NET registry

Of 483 patients registered in the national AID-NET – a clinical registry of children with autoinflammatory diseases compiled in cooperation with the GKJR – 169 patients exhibited a heterozygous phenotype. Data were analysed with respect to successful termination of colchicine therapy.

### Level of evidence and grades of recommendation

The levels of evidence and grades of recommendation were determined according to the Oxford Centre for Evidence-based Medicine and are provided in Table 1 [32]. All relevant articles were rated individually and the highest applicable rating was assigned to the corresponding statement.

**Table 1:** Consensus statements

		Statements	LoA	LoE	GR
Scope of application	1 A	Strategies of the PRO-KIND FMF project group apply for patients with clinically diagnosed FMF.	16/17	n.a.	n.a.
Diagnosis	2 A	The clinical diagnosis of FMF typically includes short-lasting recurrent fever episodes and increased inflammatory markers. Additionally, in most patients signs of serositis are present.	17/17	4	C
	2B	Genetics play an important role in the diagnosis of FMF. The interpretation of the test results has to take into account the nature of the genotype (confirmatory, consistent, inconclusive or no variant) as well as the clinical phenotype.	17/17	2a	B
	2 C	If the diagnosis is unclear, successful use of colchicine can confirm the suspected diagnosis after a sufficiently long observation period with persisting symptoms (e.g. 3–6 months).	17/17	1b-	B
	2D	A positive family history can support the clinical diagnosis of FMF.	15/15	2b	B
Differential diagnosis	3 A	In young children with non-confirmatory genotype, FMF diagnosis should be questioned and differential diagnoses such as PFAPA syndrome and age-related physiological susceptibility to infections should be considered.	16/17	3b	B
	3B	In unclear cases, further investigations (e.g. genetics) should be performed to identify other possible causes.	13/13	5	D
Treatment targets	4 A	The ultimate treatment goal in FMF is to reach complete control of unprovoked attacks, to minimise subclinical inflammation in between attacks and to prevent damage.	15/16	2b	C
	4B	Treatment response should be evaluated every 3–6 months. Treatment efficacy should be evaluated by a composite of parameters according to Fig. 1.	16/16	5	D
	4 C	The physician’s and patient’s judgment should be considered to assess the activity and severity of the disease.	16/16	5	D
Colchicine treatment	5 A	Treatment with colchicine should be started as soon as a clinical diagnosis is made due to its effective control of disease activity independently of the patient’s age.	16/16	1a-	A
	5B	A starting dose of ≤ 0.5 mg/day (for children < 5 years of age), 0.5-1.0 mg/day (for children 5–10 years of age), or 1.0-1.5 mg/day (for children > 10 years of age) should be administered orally. Colchicine dosage should be increased in a stepwise fashion (e.g. 0.25 mg or 0.5 mg/step) up to a maximum of 2.5 mg/day to control disease in patients who do not clinically respond to the standard dosage (according to Paediatrics, 2007). Colchicine application twice or three times daily may be helpful to avoid gastrointestinal adverse events.	16/16	1b	A
	5 C	The persistence of attacks or of subclinical inflammation represents an indication to increase the colchicine dose up to the maximum tolerated dosage.	16/16	2a	B
Monitoring	6 A	Response to colchicine should be monitored every 3–6 months. Evaluation should include assessment and documentation of side effects (intolerance) and toxicity as well as adherence which should be distinguished from resistance.	16/16	2b	C
	6B	Increased inflammatory markers (e.g. CRP, SAA, ESR) in the symptom-free interval can indicate subclinical inflammation and should therefore be measured regularly.	15/16	2b	C
	6 C	Monitoring of adverse events of colchicine treatment should initially be performed every 2–3 months. The monitoring interval can be extended to 6 months if the treatment is well-tolerated.	15/15	5	D
	6D	Urine analyses for the presence of proteinuria and the determination of blood pressure should be performed regularly in order to detect renal amyloidosis.	15/15	3a	C
Colchicine intolerance / inadequate colchicine response	7 A	An inadequate colchicine effect should be determined by a specialist. Before considering inadequate response, adherence to therapy should be confirmed.	15/15	3b	C
	7B	If colchicine intolerance is suspected, consider appropriate diagnostic measures to exclude other differential diagnoses.	15/15	5	D
	7 C	If there is colchicine intolerance or inadequate response, existing at least over a period of 3–6 months, the decision to intensify therapy should be made, justified and documented by a specialist.	15/15	5	D
	7D	If there is inadequate colchicine response and/or colchicine intolerance, treatment with IL-1 antagonists should be considered. The colchicine medication should be maintained.	14/14	1b	A
	7E	When choosing IL-1 antagonists, efficacy, especially in children, frequency of injections and severity of local reactions should be considered. In addition, cost-effectiveness and approval status should be taken into account.	15/15	4	C
	7 F	With respect to the treat-to-target strategy, the possibility of on-demand therapy and the possibility of dose reduction / increase in application intervals of biologics can be considered.	15/15	4	C
	7G	In the case of persistent arthritis in patients with FMF, the additional administration of non-steroidal anti-inflammatory drugs (NSAIDs), local glucocorticoids and methotrexate and, depending on disease activity, TNF inhibitors should be considered.	14/14	4	C
Colchicine reduction / termination	8 A	If a patient is stable with no attacks for more than 3 years and no elevated APR, dose reduction could be considered after expert consultation and with continued monitoring.	14/14	3b	B
	8B	In the presence of a non-confirmatory genotype including heterozygous FMF, colchicine might be terminated in asymptomatic patients. These patients should be followed up regularly.	14/14	3b	B

## Results

### Treatment targets and treatment decisions survey

The questionnaire was returned by 50% (70 out of 141) of the questioned certified paediatric rheumatologists. With respect to treatment targets, the survey addressed the following critical situations: (i) persisting subclinical inflammation in the setting of complete clinical remission, (ii) persisting attacks despite verified treatment with the maximally tolerated colchicine dose, (iii) suspected non-adherence to therapy, and (iv) colchicine intolerance.

Serum amyloid A (SAA) was rated as the most suitable biomarker to evaluate inflammation in FMF (Supplementary Figure S2A). In the case of clinical remission, most participants decided to increase the colchicine dose with persistent CRP levels of ≥ 20–30 mg/L (normal range: 0–5 mg/L) or SAA levels of ≥ 50–100 mg/L (normal range: 0-6.4 mg/L). The majority of participants regarded mean CRP levels of ≥ 30–40 mg/L or SAA levels of ≥ 100–200 mg/L as an indication to initiate biological treatment (Supplementary Figure S2B).

In a colchicine-resistant patient, an attack frequency of > 4 attacks per year was most commonly regarded as an indication for biological agents (Supplementary Figure S2C). However, a third of the participants stated that a definite threshold for the tolerable number of attacks could not be determined.

When suspecting non-adherence to colchicine therapy, the following measures were considered most appropriate: (i) serological drug monitoring, (ii) once daily intake of the medication, and (iii) plausibility check of the required vs. prescribed medication (Supplementary Figure S2D).

In a patient with clinical signs of colchicine intolerance or toxicity, several parameters prompted most participants to reduce or terminate colchicine therapy and possibly start an IL-1 antagonist: persistent diarrhoea > 2–3 times daily, leukocyte count < 2.0-2.5/nL, aspartate aminotransferase > 100–120 U/L, creatine kinase > 221–240 U/L, myalgias, and proximal muscle weakness (Supplementary Figure S2E).

### Diagnosis of FMF and treatment decisions in different clinical scenarios

In the aforementioned survey, we also asked for treatment decisions (start, adjustment and termination of colchicine, start of biological agents, other modalities) in different clinical scenarios. Thus, in a patient with concomitant headache, dizziness and weakness (case 16), 44% of paediatric rheumatologists would offer psychological support, while this number amounts to 56% in case of clear FMF symptoms, high inflammation and known non-adherence (case 17). In a patient with persistent sacroiliitis with beginning destruction and otherwise low clinical disease activity, 60% would start a TNF-α inhibitor and 21% canakinumab (case 19). 53% consider colchicine dose reduction in patients with an unclear genetic diagnosis and a symptom-free interval of 6 years (case 21).

### Data on colchicine therapy derived from the national AID-NET registry

Previously published data on colchicine dose according to age, genotype and anthropometric measures as well as the effect of dose escalation were presented during the consensus conference and were incorporated into the decision-making process [26]. Among 409 analysed patients, 3.7% (n = 15) did not show an adequate response despite the maximum tolerated dose of colchicine (2–3 mg/day) [14, 33].

A new analysis using data derived from the national AID-NET showed that colchicine therapy was terminated in 44 patients with heterozygous *MEFV* mutations. In 17 patients, colchicine was re-introduced due to recurrent symptoms. The other 27 patients (63% male, mean age at disease onset 4.84 ± 3.15 years, mean initial colchicine dosages 0.7 ± 0.32 mg/day) remained symptom-free without medication (mean follow-up 2.04 ± 1.55 years).

### Development of multidimensional treatment targets

For the development of multidimensional treatment targets, in addition to the attack frequency, the number of school or work days missed due to FMF, the level of inflammatory markers in the attack-free intervals, the occurrence of chronic sequelae, as well as the subjective patient and physician reported outcome measure of the satisfaction with the current disease status were taken into account. This grading leads to the following disease severity categories: remission or minimal disease activity, mild/moderate/severe disease activity. Further management follows the assignment into the different levels of severity (Fig. 1). A printable version of this composite score for use in clinical practice is provided in the Supplementary materials.

### Literature review

The search strategies for the single statements are provided in Supplementary Table S1. The information extracted from the relevant articles was incorporated into the preliminary statements listed in Supplementary Table S2. The online poll on these statements was completed by 17 participants and indicated sufficient agreement to proceed with the consensus conference (Supplementary Table S3).

### Statements

The developed consensus statements together with the level of agreement as well as the level of evidence and the grade of recommendation are summarised in Table 1.

Statement 1 describes for which patients the treat-to-target recommendations were developed. Patients with a genetically confirmed diagnosis of FMF without clinical symptoms and/or persistent inflammation should be closely followed up to detect disease manifestations early.

The second statement (*diagnosis*) highlights the different central aspects of the diagnosis, e.g. the presence of febrile episodes and signs of peritonitis, pleuritis and/or arthritis [2]. Pericarditis is a rare manifestation and might be associated with secondary amyloidosis [34, 35]. While different inflammation markers are frequently elevated, [26, 36] the S100 molecules seem particularly sensitive for the differentiation of FMF episodes from other febrile conditions [37].

Genetic analysis can confirm FMF diagnosis in the case of an unclear (oligosymptomatic) clinical presentation and increase the specificity of the diagnosis in patients presenting with symptoms typical of FMF.

A confirmatory genotype is defined by two bi-allelic (likely) pathogenic variants and confirms the diagnosis of FMF [38]. A consistent genotype is characterised by two (likely) pathogenic one-allelic variants or one (likely) pathogenic and one variant of uncertain significance (VUS) on two alleles, respectively. An inconclusive genotype is present in case of one (likely) pathogenic or two rare VUS. In the presence of a non-confirmatory genotype, the diagnosis can only be made in case of a clear clinical phenotype [39]. If no variant is found, the diagnosis of FMF is not supported. Amongst 316 evaluated *MEFV* variants, five were classified as ´pathogenic´ (c.2040G > A and c.2040G > C [p.Met680Ile], c.2080 A > G [p.Met694Val], c.2082G > A [p.Met694Ile], c.2177T > C [p.Val726Ala]) and 48 variants as ´likely pathogenic**´** [40, 41].

The inclusion of the response to colchicine treatment improves the sensitivity of well-established diagnostic criteria and a positive treatment response is commonly used to support the diagnosis of FMF in daily practice [42, 43]. When using colchicine as a diagnostic parameter, the possible placebo effect should be considered and the clinical course should be observed over a sufficiently long observation period [44].

The clear familial clustering indicates that the occurrence of familial cases supports the diagnosis of FMF in the index patient [2, 45–47].

The literature search for statement 3 (*differential diagnosis*) revealed mostly narrative reviews which underline the importance of considering PFAPA syndrome and recurrent viral infections as the most frequently observed differential diagnoses in this age-group [48–50].

The working group agreed that many other differential diagnoses, e.g. haemato-oncological diseases, other monogenic autoinflammatory diseases, immunodeficiencies and recurrent infections should be considered especially in case of unclear presentation and non-confirmatory genotype. Further examinations should be guided by the accompanying symptoms.

**Fig. 2:**
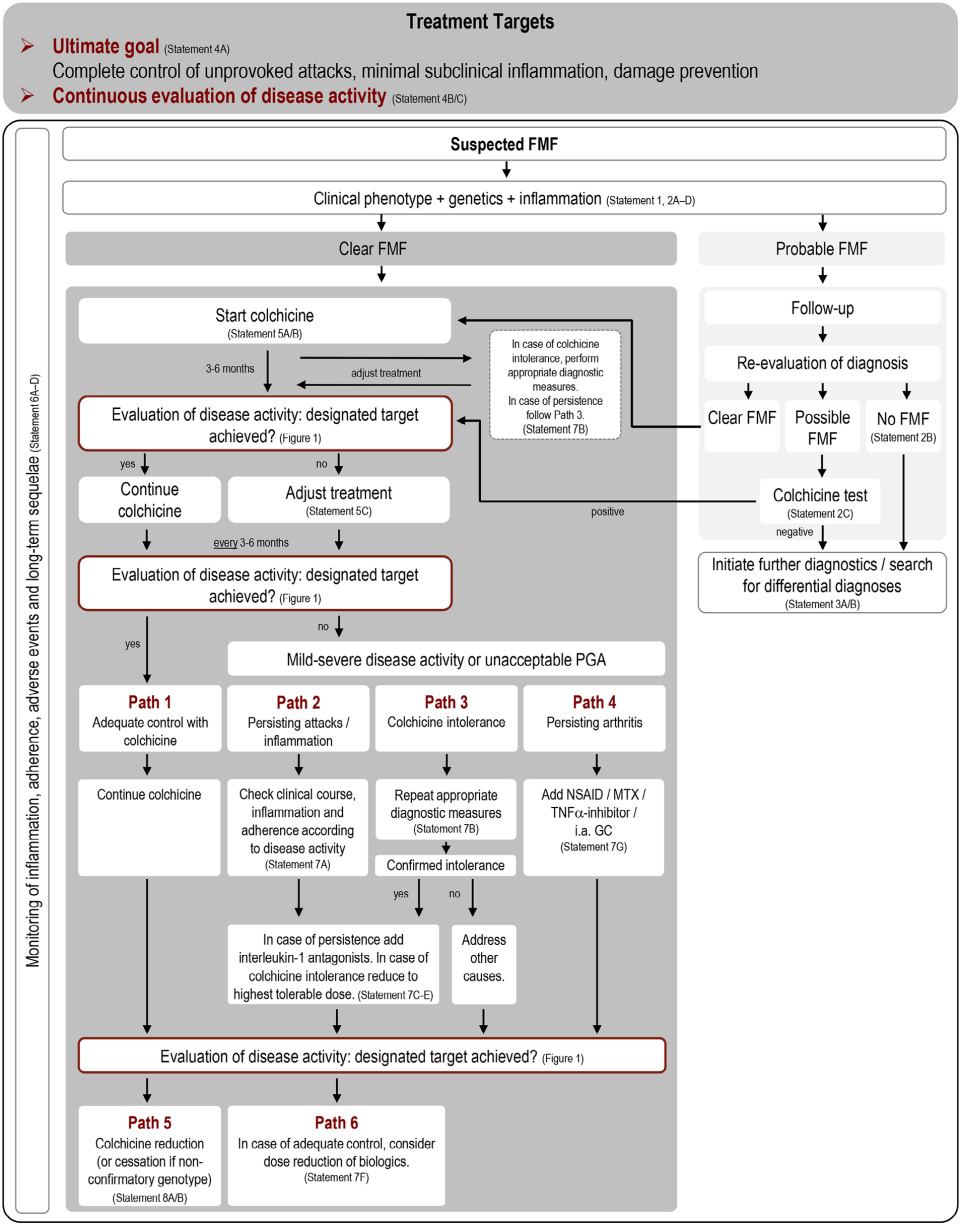
Consensus treat-to-target approach in the treatment of familial Mediterranean fever. Following diagnostic evaluation patients with clear or probable FMF should be treated according to the designated treatment path. Evaluation of disease activity is to be performed every 3–6 months according to the composite score proposed in Fig. 1. For dosing of IL-1 antagonists in children, we refer to the Summary of Product Characteristics of the chosen agent in line with the patient’s medical need. Legend: GC, glucocorticoid; MTX, methotrexate; NSAID, non-steroidal anti-inflammatory drug; PGA, Patient/Physician Global Assessment

Statement 4 (*treatment targets*) addresses the treatment goals in FMF. Persistent (sub-)clinical inflammation is a frequently observed phenomenon in FMF, [51] with SAA and the S100 molecules representing sensitive markers to detect ongoing inflammation during the attack-free intervals [52–54]. However, it is not possible to define an evidence-based threshold value for any inflammation parameter predicting the occurrence of damage during follow-up [55]. Existing assessment tools for evaluation of disease activity and severity cover different aspects, e.g. PROMs, clinical manifestation, damage, quality of life and inflammation markers (see also discussion) [29–31, 56–58]. While these scores have been widely used for assessment in both routine care and clinical trials, the members of the working group agreed that none of these instruments entirely fulfilled the requirements of a thorough target definition to be used in a treatment consensus plan. The proposed newly developed treatment targets (Fig. 2) incorporate multidimensional aspects of disease presentation including physician’s and patient’s judgment as well as chronic sequelae and allow the definition of different disease activity stages.

Statement 5 (*colchicine treatment*) focuses on the management of colchicine therapy and is primarily based on previously performed systematic literature searches [14, 27, 59, 60]. The previous recommendations are additionally supported by a recent study that proved the efficacy and safety of colchicine especially in children aged < 4 years [61].

Splitting the daily colchicine doses might reduce side effects like lactose intolerance, diarrhoea and abdominal cramps. On the other hand, a single daily dose has the same efficacy and can be helpful to increase compliance [62]. It is currently a matter of debate whether the kind of colchicine preparation, e.g. defined by the amount of (minor) alkaloids, has an impact on its side effect profile and efficacy [63].

The members of the working group agreed that lower initial colchicine dosages might be considered in heterozygous mutation carriers with a mild phenotype. However, due to the limited data available, [26, 64] no genotype-specific recommendation can be made at present.

Aspects of monitoring disease activity and adverse events are covered in statement 6 (*monitoring*). According to observational studies, side effects occur in up to a fifth of patients, frequently preventing maintenance of the effective colchicine dose [61, 65]. Therefore, non-adherence always has to be considered in the evaluation of colchicine resistance [66, 67]. The monitoring interval of 3–6 months was adopted from the EULAR recommendations [14]. In patients newly starting or escalating therapy, an intensified monitoring frequency was considered appropriate by the working group, involving the general paediatrician where applicable. With respect to colchicine toxicity, diarrhoea, leukopenia as well as liver and muscle enzymes need to be assessed [14, 61, 68].

Besides treatment safety, monitoring acute phase reactants including CRP, ESR and SAA, also in the symptom-free period, will help detect subclinical inflammation [52, 69–71]. For this purpose, phagocyte-specific S100 protein concentrations (i.e. S100A12 or the S100A8/A9 complex, also known as MRP8/14 or serum-calprotectin) are particularly specific in FMF patients [37, 54, 72].

Persistent elevations of inflammatory markers may predict the development of amyloidosis and may therefore justify treatment escalation [73, 74]. Screening measures for renal amyloidosis include routine assessment of blood pressure and proteinuria [75]. Detection of proteinuria in consecutive samples requires further diagnostic measures to confirm the diagnosis of amyloidosis or glomerulonephritis associated with IgA vasculitis and polyarteritis nodosa [76, 77].

Statement 7 (*colchicine intolerance / inadequate colchicine response*) addresses the issues of colchicine intolerance and insufficient disease control despite application of maximum tolerated colchicine dosages. In this context, the working group highlighted the importance of confirming adherence to therapy before initiating any further steps. According to the survey, preferred measures in this scenario include close monitoring of acute phase reactants as well as a thorough discussion about the prognosis (Supplementary Figure S2D). In addition, the Medication Adherence Scale for Familial Mediterranean Fever (MASIF) questions from 4 categories on compliance with colchicine can be addressed [78].

In patients presenting with diarrhoea, myalgia or abnormal leukocyte counts or transaminases, further diagnostic measures should be initiated to confirm colchicine intolerance and reduce the colchicine dose accordingly (Supplementary Figure S2E). The literature review did not yield a uniform definition of colchicine resistance [79–81]. An overview of previously published definitions is presented in Supplemental Table S4.

An inadequate treatment response should be confirmed according to the multidimensional parameters in Fig. 1 after a sufficiently long observation period (3–6 months). The efficacy of IL-1-blocking drugs in colchicine-resistant FMF (crFMF) has recently been confirmed in randomised controlled trials [18–20]. The working group recommends accompanying this treatment by the highest tolerable dose of colchicine although data on the colchicine maintenance and dosage is scarce. While comparative trials between anakinra and canakinumab are lacking, observational studies suggest comparable efficacy of both agents [82]. Due to superior controllability, anakinra was preferred in patients on haemodialysis [83–85]. Injection reactions provoked a change from anakinra to canakinumab in a number of patients [84, 85]. Several studies showed successful dose reduction or increase of application intervals of IL-1 antagonists in patients with stably controlled disease [18, 86, 87]. In patients with clear prodromes or triggers preceding their attacks, on-demand application of anakinra can be feasible [79, 88]. FMF patients with persisting arthritis often require different therapeutic agents including non-steroidal anti-inflammatory drugs, methotrexate and TNF-α inhibitors [89, 90].

Statement 8 (*colchicine reduction / termination*) discusses long-term management of colchicine in patients with well-controlled disease. Colchicine-free remission is rare, especially in M694V homozygous patients [91]. On the other hand, uncontrolled studies showed that patients with a non-confirmatory genotype or heterozygotes are more likely to successfully terminate colchicine treatment [48, 92, 93]. The working group agreed on an interval of three years without clinical or subclinical inflammation before considering colchicine reduction in patients with adequate disease control.

### Development of consensus treatment plans

Based on the derived data and literature search a consensus treatment plan was developed for patients with a definite diagnosis of FMF (Statement (S) 1 and 2). In case of probable FMF the diagnosis should be re-evaluated, other differential diagnosis considered (S 3) or a diagnostic trial of colchicine performed (S 2).

In patients with clear FMF, colchicine should be started at time of diagnosis (S 5) and achievement of the designated targets (Fig. 1) should be evaluated during follow-up (S 4 and 6). In case of sufficient disease control, patients remain in **treatment path 1** (**TP1**, *continue colchicine*) and continue colchicine treatment. If treatment targets are not achieved (physician´s and patient´s assessment and/or persistent mild to severe disease activity, S 4 and 6), patients should be accurately re-evaluated (S 7) before entering **TP2** (*persisting attacks / inflammation*), **TP3** (*colchicine intolerance*) or **TP4** (*persisting arthritis*; S 7). In patients successfully treated with biologics or colchicine, dose reduction, on-demand treatment (in case of biological treatment only) or termination can be considered according to **path 5 and 6** (*colchicine reduction* and *adjustment/reduction of biologics*, S 7, S 8) after a sufficiently long observation period.

## Discussion

The ultimate goal in the management of FMF is the prevention of attacks and long-term complications [14]. Continuous colchicine administration is the mainstay of therapy [14, 27, 60]. However, in some patients this treatment is not sufficient: recurrent attacks, continuous arthritis and/or systemic inflammation persist, long-term complications like amyloidosis develop or treatment is not tolerated due to side effects. Since previous treatment recommendations are limited by the lack of instruments for a differentiated assessment of disease severity, the developed consensus treatment plan provides multidimensional targets in order to guide personalised treatment decisions.

The treat-to-target principle pursues several overarching objectives: prevention of long-term damage, tailored treatment adjustment (i.e. avoidance of over- and undertreatment), and a target- and therefore patient-centred approach. The strategy was initially introduced in the management of hypertension and diabetes mellitus where numerical targets were set [94, 95]. As discussed above, the definition of a uniform treatment target comprehensively capturing disease activity of chronic inflammatory diseases is more challenging and discussions on the most suitable set of parameters are ongoing [96]. The lack of comparative studies evaluating the application of different treatment targets with respect to patient satisfaction and long-term outcomes represents an additional challenge in this context. A prospective analysis of the long-term impact of the T2T approach will be desirable in the future.

The introduction of a T2T strategy in the treatment of FMF is intended to benefit all patients. In patients unresponsive to colchicine, especially, the T2T approach will help swift and well-monitored treatment escalation to prevent long-term damage. Currently, the estimated number of colchicine-resistant patients amounts to approximately 5% [16]. Fifteen (3.7%) out of 409 patients extracted from the German AID-NET registry fulfilled the definition of colchicine resistance [26]. Besides non-responsive patients, the initiative aims to improve overall patient care by providing a more comprehensive target description that we consider superior and more patient-focussed than the existing definitions of colchicine resistance that currently guide the use of biological agents [18, 97]. Finally, the T2T approach is designed to make treatment decisions more transparent: By including the patient’s assessment and presenting the available treatment paths, the strategy aims to be comprehensible to the patient population and thereby promote adherence to therapy [98]. The suggested treatment strategy is vastly based on and in line with previously published recommendations for the management of FMF (Supplemental Table S1) [14, 27]. Compared to the EULAR recommendations, this T2T approach was developed with a focus on management in children. The core difference is the central importance of the newly developed score of multidimensional treatment targets guiding therapeutic decisions throughout.

Previously, different assessment tools have been proposed to capture disease activity and degree of damage. These tools are of great value to assess disease characteristics they were designed to capture. However, several aspects render them unsuitable for the application in the context of a T2T approval: They (i) are rather oligodimensional, [58] (ii) do not include the acute phase response or PROMs, [57] or (iii) capture primarily improvement under treatment [57, 58, 99, 100]. Other scores focus on the quality of life, chronic sequelae and response to treatment [29, 30, 101].

The Autoinflammatory Disease Activity Index (AIDAI) is based on dichotomous recording of individual disease parameters over one month [56]. Since the parameters are recorded by the patient and/or the parents, this instrument particularly reflects the subjective assessment. The International Severity Score for FMF (ISSF) consists of ten items, three of which capture chronic sequelae. Since the physician´s global assessment was used to define a gold standard, this item - as well as other PROMs - were not included in the score [31].

To overcome the limitations of previously proposed assessment tools, different aspects of the published instruments were combined in order to define user-friendly multidimensional treatment targets (Fig. 1). The validation of this novel composite in a large patient cohort in comparison with other established assessment tools would be desirable in the future.

The recently published definitions for colchicine resistance were mainly developed to define the indication for introducing an IL-1-targeting treatment approach. They pay particular attention to the frequency of attacks (between > 4 and ≥ 24 attacks / year according to different authors) as well as the presence of persisting (sub-) inflammation [97, 102]. Since attacks can occur with varying intensity and thus affect the quality of life to various degrees in different individuals, we complemented the frequency of attacks (**item 1**) with additional items. We therefore propose a scoring system that captures different aspects reflecting disease activity.

A child´s involvement in daily activity allowing regular physical, social and mental development is a prominent goal in the management of chronic conditions [103]. Since the number of missed school days is an easy parameter to collect, we have included this item as a measure of social participation (**item 2**). Similarly, the number of missed work days was included as a measure of social participation in older adolescents and adults. While this initiative was led by a group of paediatric rheumatologists, we believe that the score is equally suitable in the adult patient population.

(Sub-)clinical inflammation (**item 3**) directly affects patients’ well-being and is a prerequisite for many chronic sequelae, e.g. amyloidosis and growth retardation [74, 104]. Since there is no published evidence on the threshold values of inflammation that may be tolerated, [102, 105] the ranges given in the table are based on the results of the survey and the discussion within the working group (**item 3**). Ideally, complete control of inflammatory parameters is sought, but potential therapeutic side effects must be considered when intensifying treatment.

Although chronic sequelae (**item 4**) are the result of persistent inflammation in the past and thus do not capture current disease activity, this element was included in the multidimensional treatment goals since it correlates with the individual predisposition for damage. Progression of sequelae must be avoided in already affected patients. The occurrence of secondary damage is also co-determined by known polymorphisms in other genes (e.g. SAA) as well as unknown factors whose complexity cannot be captured in a treatment plan [106, 107].

Due to the challenges in assessing disease activity and the subjective perception of disease activity, a question on satisfaction with the health status was included in the list of target items (**item 5**). Of note, especially in case of minimal and mild disease activity the judgement by the physician and/or patient and caregiver may prompt a change in management, e.g. increase of colchicine or introduction of anti-cytokine treatment.

The proposed multidimensional target approach provides a system to categorise disease activity as follows: remission/minimal, mild, moderate and severe. Accordingly, a recommendation is made to adjust the patient´s management as indicated in Figs. 1 and 2.

The majority of patients with an uncomplicated course of FMF are well-controlled with colchicine therapy (**TP1**) [14, 27, 60]. Based on recent data, reduction or discontinuation of colchicine therapy can be successful, especially in the presence of a non-confirmatory phenotype; [48, 91–93, 108] such an approach is represented by **TP5**. However, this approach should only be carried out under careful clinical and laboratory examination.

In cases of persistent inflammation and/or attacks, inflammation and adherence should be closely monitored over an extended period of time before initiating therapy with an IL-1-blocking drug (**TP2**) [18–20, 27, 109, 110]. In the presence of side effects to colchicine, it is also advisable to confirm intolerance by thorough evaluation before initiating a change in the therapeutic regimen (**TP3**) [27, 68, 80]. Persistent arthritis may result in the need for a well-established antirheumatic therapy (**TP4**) [89, 90, 111]. Recent studies also imply, that an anti-IL1-directed therapy can be effective in FMF-associated arthritis [112]. If treatment goals are achieved through the introduction of an anti-cytokine approach, elongation of application intervals, dosage de-escalation or on-demand treatment can be considered (**TP6**) [18, 79, 84, 86, 88, 113, 114]. In the latter case, short-acting drugs (e.g. anakinra) can be applied at the onset of an attack or long-acting drugs (e.g. canakinumab) at the return of clinical signs or an increase in inflammatory parameters after weeks to months.

For the development of the T2T treatment strategy, we considered therapeutic agents for which sufficient evidence is available. The drugs listed below have shown efficacy in observational studies, but further controlled studies are pending. Two retrospective cohort studies described an improvement in pre-existing amyloidosis and a reduction in attack frequency in patients with FMF treated with tocilizumab [21, 115]. Efficacy of this drug in FMF was also supported by a recent randomized placebo-controlled phase II study [116]. In case of persisting symptoms despite orally applied maximal tolerated colchicine dosages, patients might benefit from weekly intravenous colchicine application [117–119]. However, it must be taken into consideration that severe side effects can occur, especially in the case of incorrect dosing [120]. Furthermore, single reports described the use of the JAK-inhibitor tofacitinib in patients with inadequate response to anti-cytokine treatment or co-existing rheumatoid arthritis [121, 122]. It has yet to be seen whether the evidence for the use of these drugs in FMF and their long-term effects will improve in the future, allowing them to be included in treatment recommendations.

## Conclusion

The proposed consensus treatment plan for the management of FMF incorporates multidimensional targets allowing transparent treatment decisions, which will promote personalised disease management and increases adherence to therapy. The reliability of the proposed target definitions for the detection of disease severity will be the subject of further studies.

### Electronic supplementary material

Below is the link to the electronic supplementary material.

Supplementary Material 1

## Data Availability

Not applicable.

## List of abbreviations

crFMF: Colchicine-resistant familial Mediterranean fever
CRP: C-reactive protein
FMF: Familial mediterranean mever
GKJR: German Society for Paediatric Rheumatology
IL: Interleukin
MEFV: Mediterranean fever
PROMs: Patient-reported outcome measures
SAA: Serum amyloid A
T2T: Treat-to-target
TP: Treatment path
VUS: Variant of unknown significance

## References

[1] Schwartz DM, Kitakule MM, Dizon BL (2021). Systematic evaluation of nine monogenic autoinflammatory diseases reveals common and disease-specific correlations with allergy-associated features. Ann Rheum Dis Published Online First: 22 February.

[2] Sohar E, Gafni J, Pras M (1967). Familial Mediterranean fever. A survey of 470 cases and review of the literature. Am J Med.

[3] Touitou I, Sarkisian T, Medlej-Hashim M (2007). Country as the primary risk factor for renal amyloidosis in familial Mediterranean fever. Arthritis Rheum.

[4] Ancient missense mutations (1997). In a new member of the RoRet gene family are likely to cause familial Mediterranean fever. Int FMF Consortium Cell.

[5] Chae JJ, Wood G, Masters SL (2006). The B30.2 domain of pyrin, the familial Mediterranean fever protein, interacts directly with caspase-1 to modulate IL-1beta production. Proc Natl Acad Sci U S A.

[6] Park YH, Remmers EF, Lee W (2020). Ancient familial Mediterranean fever mutations in human pyrin and resistance to Yersinia pestis. Nat Immunol.

[7] Lainka E, Bielak M, Lohse P (2012). Familial Mediterranean fever in Germany: epidemiological, clinical, and genetic characteristics of a pediatric population. Eur J Pediatr.

[8] Ozen S, Karaaslan Y, Ozdemir O (1998). Prevalence of juvenile chronic arthritis and familial Mediterranean fever in Turkey: a field study. J Rheumatol.

[9] Sarkisian T, Ajrapetian H, Beglarian A et al. Familial Mediterranean Fever in armenian population. Georgian Med News 2008;:105–11. 18403822

[10] Yasar Bilge NS, Sari I, Solmaz D (2018). Comparison of early versus late onset familial Mediterranean fever. Int J Rheum Dis.

[11] Tanatar A, Karadağ ŞG, Çakan M (2021). Age of onset as an influencing factor for disease severity in children with familial Mediterranean fever. Mod Rheumatol.

[12] Gezgin Yildirim D, Gönen S, Fidan K (2020). Does Age at Onset affect the clinical presentation of familial Mediterranean Fever in Children?. J Clin Rheumatol Published Online First: 24 November.

[13] Özdel S, Özçakar ZB, Kunt S (2016). Late-onset disease is associated with a mild phenotype in children with familial Mediterranean fever. Clin Rheumatol.

[14] Ozen S, Demirkaya E, Erer B (2016). EULAR recommendations for the management of familial Mediterranean fever. Ann Rheum Dis.

[15] Zemer D, Livneh A, Danon YL (1991). Long-term colchicine treatment in children with familial mediterranean fever. Arthr Rhuem.

[16] Majeed HA, Barakat M (1989). Familial mediterranean fever (recurrent hereditary polyserositis) in children: analysis of 88 cases. Eur J Pediatr.

[17] Lachmann HJ. Long-Term Complications of Familial Mediterranean Fever. In: Gattorno M, ed. Familial Mediterranean Fever. Cham:: Springer International Publishing 2015. 91–105. doi:10.1007/978-3-319-14615-7_6.

[18] De Benedetti F, Gattorno M, Anton J (2018). Canakinumab for the treatment of Autoinflammatory recurrent fever syndromes. N Engl J Med.

[19] Ozen S, Ben-Cherit E, Foeldvari I (2020). Long-term efficacy and safety of canakinumab in patients with colchicine-resistant familial Mediterranean fever: results from the randomised phase III CLUSTER trial. Ann Rheum Dis.

[20] Ben-Zvi I, Kukuy O, Giat E (2017). Anakinra for Colchicine-Resistant Familial Mediterranean Fever: a Randomized, Double-Blind, placebo-controlled trial. Arthritis Rheumatol.

[21] Colak S, Tekgoz E, Cinar M (2021). The assessment of tocilizumab therapy on recurrent attacks of patients with familial Mediterranean fever: a retrospective study of 15 patients. Mod Rheumatol.

[22] Özçakar ZB, Yüksel S, Ekim M (2012). Infliximab therapy for familial Mediterranean fever-related amyloidosis: case series with long term follow-up. Clin Rheumatol.

[23] Hinze CH, Oommen PT, Dressler F (2018). Development of practice and consensus-based strategies including a treat-to-target approach for the management of moderate and severe juvenile dermatomyositis in Germany and Austria. Pediatr Rheumatol Online J.

[24] Ravelli A, Consolaro A, Horneff G (2018). Treating juvenile idiopathic arthritis to target: recommendations of an international task force. Ann Rheum Dis.

[25] Klein A, Minden K, Hospach A (2020). Treat-to-target study for improved outcome in polyarticular juvenile idiopathic arthritis. Ann Rheum Dis.

[26] Knieper AM, Klotsche J, Lainka E (2017). Familial Mediterranean fever in children and adolescents: factors for colchicine dosage and predicting parameters for dose increase. Rheumatology (Oxford).

[27] Kallinich T, Blank N, Braun T (2019). [Evidence-based treatment recommendations for familial Mediterranean fever: a joint statement by the Society for Pediatric and adolescent rheumatology and the german society for Rheumatology]. Z Rheumatol.

[28] Piram M, Koné-Paut I, Lachmann HJ (2014). Validation of the auto-inflammatory diseases activity index (AIDAI) for hereditary recurrent fever syndromes. Ann Rheum Dis.

[29] Ozen S, Demirkaya E, Duzova A (2014). FMF50: a score for assessing outcome in familial Mediterranean fever. Ann Rheum Dis.

[30] Konukbay D, Gattorno M, Yildiz D (2016). A novel assessment tool for clinical care of patients with autoinflammatory disease: juvenile autoinflammatory disease multidimensional assessment report. Clin Exp Rheumatol.

[31] Demirkaya E, Acikel C, Hashkes P (2016). Development and initial validation of international severity scoring system for familial Mediterranean fever (ISSF). Ann Rheum Dis.

[32] Oxford Centre for Evidence-Based Medicine. : Levels of Evidence (March 2009). https://www.cebm.ox.ac.uk/resources/levels-of-evidence/oxford-centre-for-evidence-based-medicine-levels-of-evidence-march-2009.

[33] Hentgen V, Grateau G, Kone-Paut I (2013). Evidence-based recommendations for the practical management of familial Mediterranean Fever. Semin Arthritis Rheum.

[34] Alsarah A, Alsara O, Laird-Fick HS (2017). Cardiac manifestations of familial Mediterranean fever. Avicenna J Med.

[35] Erken E (2018). Cardiac disease in familial Mediterranean fever. Rheumatol Int.

[36] Lachmann HJ, Sengül B, Yavuzşen TU (2006). Clinical and subclinical inflammation in patients with familial Mediterranean fever and in heterozygous carriers of MEFV mutations. Rheumatology (Oxford).

[37] Kallinich T, Wittkowski H, Keitzer R (2010). Neutrophil-derived S100A12 as novel biomarker of inflammation in familial Mediterranean fever. Ann Rheum Dis.

[38] Shinar Y, Obici L, Aksentijevich I (2012). Guidelines for the genetic diagnosis of hereditary recurrent fevers. Ann Rheum Dis.

[39] Ozen S, Bilginer Y (2014). A clinical guide to autoinflammatory diseases: familial Mediterranean fever and next-of-kin. Nat Rev Rheumatol.

[40] Van Gijn ME, Ceccherini I, Shinar Y (2018). New workflow for classification of genetic variants’ pathogenicity applied to hereditary recurrent fevers by the International Study Group for systemic Autoinflammatory Diseases (INSAID). J Med Genet.

[41] Gangemi S, Manti S, Procopio V (2018). Lack of clear and univocal genotype-phenotype correlation in familial Mediterranean fever patients: a systematic review. Clin Genet.

[42] Demirkaya E, Saglam C, Turker T (2016). Performance of different diagnostic criteria for familial Mediterranean Fever in children with periodic fevers: results from a Multicenter International Registry. J Rheumatol.

[43] La Regina M, Ben-Chetrit E, Gasparyan AY (2013). Current trends in colchicine treatment in familial Mediterranean fever. Clin Exp Rheumatol.

[44] Ozaltin F, Bilginer Y, Gülhan B (2014). Diagnostic validity of colchicine in patients with familial Mediterranean fever. Clin Rheumatol.

[45] Heller H, Sohar E, Gafni J (1961). Amyloidosis in familial Mediterranean fever. An independent genetically determined character. Arch Intern Med.

[46] Al-Wahadneh AM, Dahabreh MM (2006). Familial Mediterranean fever in children: a single centre experience in Jordan. East Mediterr Health J.

[47] Ozel AM, Demirtürk L, Yazgan Y (2000). Familial Mediterranean fever. A review of the disease and clinical and laboratory findings in 105 patients. Dig Liver Dis.

[48] Hentgen V, Grateau G, Stankovic-Stojanovic K (2013). Familial Mediterranean fever in heterozygotes: are we able to accurately diagnose the disease in very young children?. Arthritis Rheum.

[49] Soriano A, Soriano M, Espinosa G (2020). Current therapeutic options for the Main Monogenic Autoinflammatory Diseases and PFAPA Syndrome: evidence-based Approach and proposal of a practical guide. Front Immunol.

[50] Manna R, Rigante D (2019). Familial Mediterranean Fever: assessing the overall clinical impact and formulating treatment plans. Mediterr J Hematol Infect Dis.

[51] Atas N, Armagan B, Bodakci E (2020). Familial Mediterranean fever is associated with a wide spectrum of inflammatory disorders: results from a large cohort study. Rheumatol Int.

[52] Çakan M, Karadağ ŞG, Tanatar A (2021). The value of serum amyloid A levels in familial Mediterranean Fever to identify occult inflammation during asymptomatic periods. J Clin Rheumatol.

[53] Lieber M, Kallinich T, Lohse P (2015). Increased serum concentrations of neutrophil-derived protein S100A12 in heterozygous carriers of MEFV mutations. Clin Exp Rheumatol.

[54] Gohar F, Orak B, Kallinich T (2016). Correlation of secretory activity of neutrophils with genotype in patients with familial Mediterranean Fever. Arthritis Rheumatol.

[55] Erer B, Demirkaya E, Ozen S (2016). What is the best acute phase reactant for familial Mediterranean fever follow-up and its role in the prediction of complications? A systematic review. Rheumatol Int.

[56] Piram M, Frenkel J, Gattorno M et al. A preliminary score for the assessment of disease activity in hereditary recurrent fevers: results from the AIDAI (Auto-Inflammatory Diseases Activity Index) Consensus Conference. *Ann Rheum Dis* 2011;70:309–14. doi:10.1136/ard.2010.132613. 10.1136/ard.2010.132613 PMC3463866 21081528

[57] Pras E, Livneh A, Balow JE Jr, et al. Clinical differences between north african and iraqi Jews with familial Mediterranean fever. Am J Med Genet. 1998;75:216–9. 10.1002/(sici)1096-8628(19980113)75:2<216::aid-ajmg20>3.0.co;2-r. 10.1002/(sici)1096-8628(19980113)75:2<216::aid-ajmg20>3.0.co;2-r 9450890

[58] Mor A, Shinar Y, Zaks N (2005). Evaluation of disease severity in familial Mediterranean fever. Semin Arthritis Rheum.

[59] Sahr T, Kiltz U, Weseloh C (2020). [Results of the systematic literature search as basis for the ‘Evidence-based treatment recommendations for familial Mediterranean fever patients with insufficient response or intolerability to colchicine’ of the Society for Pediatric and adolescent rheumatology and the german society for Rheumatology]. Z Rheumatol.

[60] Kallinich T, Haffner D, Niehues T (2007). Colchicine use in children and adolescents with familial Mediterranean fever: literature review and consensus statement. Pediatrics.

[61] Goldberg O, Levinsky Y, Peled O (2019). Age dependent safety and efficacy of colchicine treatment for familial mediterranean fever in children. Semin Arthritis Rheum.

[62] Polat A, Acikel C, Sozeri B (2016). Comparison of the efficacy of once- and twice-daily colchicine dosage in pediatric patients with familial Mediterranean fever–a randomized controlled noninferiority trial. Arthritis Res Ther.

[63] Baglan E, Ozdel S, Bulbul M (2021). Do all colchicine preparations have the same effectiveness in patients with familial Mediterranean fever?. Mod Rheumatol.

[64] Marek-Yagel D, Berkun Y, Padeh S (2009). Clinical disease among patients heterozygous for familial Mediterranean fever. Arthritis Rheum.

[65] Satiş H, Armağan B, Bodakçi E (2020). Colchicine intolerance in FMF patients and primary obstacles for optimal dosing. Turk J Med Sci.

[66] Corsia A, Georgin-Lavialle S, Hentgen V (2017). A survey of resistance to colchicine treatment for french patients with familial Mediterranean fever. Orphanet J Rare Dis.

[67] Tekgöz E, Çolak S, Çinar FI, et al. Non-adherence to colchicine treatment is a common misevaluation in familial Mediterranean fever. Turk J Med Sci Published Online First: 7. May 2021. 10.3906/sag-2102-328. 10.3906/sag-2102-328 33957721

[68] Sag E, Bayindir Y, Adiguzel A (2020). Colchicine and Leukopenia: clinical implications. J Pediatr.

[69] Türkmenoğlu Y, Güney E, Bezen D (2021). Evaluation of S100A12 protein levels in children with familial Mediterranean fever. Turk J Med Sci.

[70] Barut K, Sahin S, Adrovic A (2018). Familial Mediterranean fever in childhood: a single-center experience. Rheumatol Int.

[71] Bayram MT, Çankaya T, Bora E (2015). Risk factors for subclinical inflammation in children with familial Mediterranean fever. Rheumatol Int.

[72] Yamasaki Y, Takei S, Imanaka H (2019). S100A12 and vascular endothelial growth factor can differentiate Blau syndrome and familial Mediterranean fever from systemic juvenile idiopathic arthritis. Clin Rheumatol.

[73] Varan O, Kucuk H, Babaoglu H (2019). Chronic inflammation in adult familial Mediterranean fever patients: underlying causes and association with amyloidosis. Scand J Rheumatol.

[74] Lachmann HJ, Goodman HJ, Gilbertson JA (2007). Natural history and outcome in systemic AA amyloidosis. N Engl J Med.

[75] Papa R, Lachmann HJ, Secondary AA, Amyloidosis (2018). Rheumatic Disease Clinics of North America.

[76] de Asúa DR, Costa R, Galván JM (2014). Systemic AA amyloidosis: epidemiology, diagnosis, and management. CLEP.

[77] Siligato R, Gembillo G, Calabrese V (2021). Amyloidosis and glomerular Diseases in Familial Mediterranean Fever. Med (Kaunas).

[78] Yesilkaya S, Acikel C, Fidanci BE et al. Development of a medication adherence scale for familial Mediterranean fever (MASIF) in a cohort of turkish children.;:7. 26393894

[79] Sag E, Akal F, Atalay E (2020). Anti-IL1 treatment in colchicine-resistant paediatric FMF patients: real life data from the HELIOS registry. Rheumatology (Oxford).

[80] Kisla Ekinci RM, Balci S, Dogruel D (2019). Canakinumab in Children with Familial Mediterranean Fever: a Single-Center, retrospective analysis. Paediatr Drugs.

[81] Ozen S, Kone-Paut I, Gül A (2017). Colchicine resistance and intolerance in familial mediterranean fever: definition, causes, and alternative treatments. Semin Arthritis Rheum.

[82] Şahin A, Derin ME, Albayrak F (2020). Assessment of effectiveness of anakinra and canakinumab in patients with colchicine-resistant/unresponsive familial Mediterranean fever. Adv Rheumatol.

[83] Fayand A, Savey L, Ducharme-Bénard S (2021). Prescription of interleukin-1 inhibitors in a french adult cohort of familial Mediterranean fever. Eur J Intern Med.

[84] Köhler BM, Lorenz H-M, Blank N (2018). IL1-blocking therapy in colchicine-resistant familial Mediterranean fever. Eur J Rheumatol.

[85] Ugurlu S, Ergezen B, Egeli BH (2020). Safety and efficacy of anti-interleukin-1 treatment in 40 patients, followed in a single centre, with AA amyloidosis secondary to familial Mediterranean fever. Rheumatology (Oxford).

[86] Eren Akarcan S, Dogantan S, Edeer Karaca N (2020). Successful management of colchicine resistant familial Mediterranean fever patients with a standardized canakinumab treatment protocol: a case series and literature review. Rheumatol Int.

[87] Babaoglu H, Varan O, Kucuk H (2020). Effectiveness of Canakinumab in Colchicine- and Anakinra-Resistant or -intolerant adult familial Mediterranean Fever Patients: a Single-Center Real-Life Study. J Clin Rheumatol.

[88] Babaoglu H, Varan O, Kucuk H (2019). On demand use of anakinra for attacks of familial Mediterranean fever (FMF). Clin Rheumatol.

[89] Langevitz P, Livneh A, Zemer D (1997). Seronegative spondyloarthropathy in familial Mediterranean fever. Semin Arthritis Rheum.

[90] Sakallioglu O, Duzova A, Ozen S (2006). Etanercept in the treatment of arthritis in a patient with familial Mediterranean fever. Clin Exp Rheumatol.

[91] Ben-Zvi I, Krichely-Vachdi T, Feld O (2014). Colchicine-free remission in familial Mediterranean fever: featuring a unique subset of the disease-a case control study. Orphanet J Rare Dis.

[92] Sönmez HE, Batu ED, Bilginer Y (2017). Discontinuing colchicine in symptomatic carriers for MEFV (Mediterranean FeVer) variants. Clin Rheumatol.

[93] Butbul Aviel Y, Rawan S, Fahoum S (2021). Discontinuation of Colchicine Therapy in Children with Familial Mediterranean Fever. J Rheumatol Published Online First: 15 May.

[94] Hansson L, Zanchetti A, Carruthers SG (1998). Effects of intensive blood-pressure lowering and low-dose aspirin in patients with hypertension: principal results of the hypertension optimal treatment (HOT) randomised trial. HOT Study Group. Lancet.

[95] Nathan DM, Genuth S, Diabetes Control and Complications Trial Research Group (1993). The effect of intensive treatment of diabetes on the development and progression of long-term complications in insulin-dependent diabetes mellitus. N Engl J Med.

[96] Mankia K, Gul H, Emery P (2021). Treating rheumatoid arthritis to an imaging target produces better outcomes, or does it?. Rheumatology.

[97] Özen S, Sag E, Ben-Chetrit E et al. Defining colchicine resistance/intolerance in patients with familial Mediterranean fever: a modified-Delphi consensus approach. *Rheumatology (Oxford)* Published Online First: 17 December 2020. doi:10.1093/rheumatology/keaa863. 10.1093/rheumatology/keaa863 33331943

[98] Benham H, Rutherford M, Kirby S (2019). Treat-to-target in rheumatoid arthritis: evaluating the patient perspective using the patient Opinion Real-Time Anonymous Liaison system: the RA T2T PORTAL study. Int J Rheum Dis.

[99] Kalkan G, Demirkaya E, Acikel CH (2012). Evaluation of the current disease severity scores in paediatric FMF: is it necessary to develop a new one?. Rheumatology (Oxford).

[100] Ozen S, Aktay N, Lainka E (2009). Disease severity in children and adolescents with familial Mediterranean fever: a comparative study to explore environmental effects on a monogenic disease. Ann Rheum Dis.

[101] Ter Haar NM, van Delft ALJ, Annink KV (2018). In silico validation of the autoinflammatory disease damage index. Ann Rheum Dis.

[102] Erden A, Batu ED, Sarı A (2018). Which definition should be used to determine colchicine resistance among patients with familial Mediterranean fever?. Clin Exp Rheumatol.

[103] Imms C, Adair B, Keen D (2016). Participation’: a systematic review of language, definitions, and constructs used in intervention research with children with disabilities. Dev Med Child Neurol.

[104] Kişla Ekinci RM, Balci S, Akay E (2019). Disease severity and genotype affect physical growth in Children with Familial Mediterranean Fever. Arch Rheumatol.

[105] Stankovic Stojanovic K, Hentgen V, Fellahi S (2017). Concordance between CRP and SAA in familial Mediterranean fever during attack-free period: a study of 218 patients. Clin Biochem.

[106] Gershoni-Baruch R, Brik R, Zacks N (2003). The contribution of genotypes at the MEFV and SAA1 loci to amyloidosis and disease severity in patients with familial Mediterranean fever. Arthritis Rheum.

[107] Cazeneuve C, Ajrapetyan H, Papin S (2000). Identification of MEFV-independent modifying genetic factors for familial Mediterranean fever. Am J Hum Genet.

[108] Tanatar A, Karadağ Ş G, Sönmez HE (2019). Short-term follow-up results of children with familial Mediterranean fever after cessation of colchicine: is it possible to quit?. Rheumatology (Oxford).

[109] Akar S, Cetin P, Kalyoncu U (2018). Nationwide experience with off-label use of Interleukin-1 Targeting Treatment in Familial Mediterranean Fever Patients. Arthritis Care Res (Hoboken).

[110] Ugurlu S, Ergezen B, Egeli BH (2021). Anakinra treatment in patients with familial Mediterranean fever: a single-centre experience. Rheumatology (Oxford).

[111] Yıldırım DG, Fidan HK, Gönen S (2020). Sacroiliitis associated with familial Mediterranean fever in childhood: a case series and review of literature. Turk J Pediatr.

[112] Kehribar DY, Özgen M (2021). Efficacy of anti-interleukin-1 treatment in colchicine-resistant arthritis in patients with familial Mediterranean fever. Eur J Rheumatol.

[113] Hentgen V, Koné-Paut I, Belot A (2020). Long-term Follow-Up and optimization of Interleukin-1 inhibitors in the management of Monogenic Autoinflammatory Diseases: Real-Life Data from the JIR Cohort. Front Pharmacol.

[114] Kurt T, Aydın F, Nilüfer Tekgöz P (2020). Effect of anti-interleukin-1 treatment on quality of life in children with colchicine-resistant familial Mediterranean fever: a single-center experience. Int J Rheum Dis.

[115] Ugurlu S, Hacioglu A, Adibnia Y (2017). Tocilizumab in the treatment of twelve cases with aa amyloidosis secondary to familial mediterranean fever. Orphanet J Rare Dis.

[116] Henes JC, Saur S, Kofler DM (2022). Tocilizumab for the treatment of familial Mediterranean Fever—A Randomized, Double-Blind, placebo-controlled phase II study. J Clin Med.

[117] Lidar M, Kedem R, Langevitz P (2003). Intravenous colchicine for treatment of patients with familial Mediterranean fever unresponsive to oral colchicine. J Rheumatol.

[118] Tal R, Semo Oz R, Amarilyo G (2020). Safety and efficacy of intravenous colchicine in children with familial Mediterranean Fever. Rheumatol Int.

[119] Rozenbaum M, Boulman N, Feld J et al. Intravenous colchicine treatment for six months: adjunctive therapy in familial Mediterranean fever (FMF) unresponsive to oral colchicine. In: *Clin Exp Rheumatol*. Italy: 2009. S105. 19796546

[120] Deaths from (2007). Intravenous colchicine resulting from a compounding pharmacy error–oregon and Washington, 2007. MMWR Morb Mortal Wkly Rep.

[121] Karadeniz H, Güler AA, Atas N (2020). Tofacitinib for the treatment for colchicine-resistant familial Mediterranean fever: case-based review. Rheumatol Int.

[122] Gök K, Cengiz G, Erol K (2017). Tofacitinib suppresses disease activity and febrile attacks in a patient with coexisting rheumatoid arthritis and familial Mediterranean fever. Acta Reumatol Port.

