# Supplementary material for: Treat-to-target strategies for the management of familial Mediterranean Fever in children
Source: Pediatr Rheumatol Online J. 2023 Sep 26;21:108. doi: 10.1186/s12969-023-00875-y (PMC10521475; doi:10.1186/s12969-023-00875-y)
Supplement: Supplementary file 1 — Supplementary Material 1 [file 12969_2023_875_MOESM1_ESM.pdf]

Composite Score to assess disease activity in patients with Familial Mediterranean Fever

**Frequency of attacks**	☐ ≤2/year		☐ 3-6/year		☐ 7-11/year	☐ ≥12/year
**School/work days missed due to FMF**	☐ ≤ 5/year		☐ 6-10/year		☐ 11-20/year	☐ >20/year
**Increased acute-phase reactants during the attack-free period**[1]	☐ CRP ≤2x UL<br>☐ SAA ≤14 mg/L		☐ CRP >2 and ≤4x UL<br>☐ SAA >14 and ≤50 mg/L		☐ CRP >4 and ≤8x UL<br>☐ SAA >50 and ≤100 mg/L	☐ CRP >8 x UL<br>☐ SAA >100 mg/L
**Chronic sequelae**[2]					☐ new or worsening sequelae	
**Evaluation**	☒ for all items<br><br>**Remission or minimal disease activity**		☒ for ≥1 item<br><br>**Mild disease activity**		☒ for ≥1 item<br><br>**Moderate disease activity**	☒ for ≥1 item<br><br>**Severe disease activity**
**Global assessment: target reached?**						
**Physician**[3]	☐ yes	☐ no	☐ yes	☐ no		
**Patient**[4]	☐ yes	☐ no	☐ yes	☐ no		
**Consequences**	**Path 1**<br>*Consider paths 5/6 if applicable*	**Path 1: observe closely**<br>*Paths 2/3 might be considered*		**Paths 2-4**		

1 ≥2 weeks after the last attack, measured at least two times 1 month apart

2 Amyloidosis, growth retardation (<3rd percentile), symptomatic anaemia requiring therapy, thrombopenia due to splenomegaly, persisting arthritis lasting > 6 weeks

3 Are you satisfied with the current FMF-related health status of your patient?

4 Is the patient satisfied with their current FMF-related health status?
